# Fused Deposition Modeling Parameter Optimization for Cost-Effective Metal Part Printing

**DOI:** 10.3390/polym14163264

**Published:** 2022-08-10

**Authors:** Claudio Tosto, Jacopo Tirillò, Fabrizio Sarasini, Claudia Sergi, Gianluca Cicala

**Affiliations:** 1 Department of Civil Engineering and Architecture, University of Catania, Viale Andrea Doria 6, 95125 Catania, Italy; 2 Department of Chemical Engineering Materials Environment, Sapienza-Università di Roma and UdR INSTM, Via Eudossiana 18, 00184 Roma, Italy

**Keywords:** additive manufacturing, fused filament fabrication, optimization, stainless steel, 3D printing

## Abstract

Metal 3D-printed parts are critical in industries such as biomedical, surgery, and prosthetics to create tailored components for patients, but the costs associated with traditional metal additive manufacturing (AM) techniques are typically prohibitive. To overcome this disadvantage, more cost-effective manufacturing processes are needed, and a good approach is to combine fused deposition modeling (FDM) with debinding-sintering processes. Furthermore, optimizing the printing parameters is required to improve material density and mechanical performance. The design of experiment (DoE) technique was used to evaluate the impact of three printing factors, namely nozzle temperature, layer thickness, and flow rate, on the tensile and bending properties of sintered 316L stainless steel in this study. Green and sintered samples were morphologically and physically characterized after printing, and the optimal printing settings were determined by statistical analysis, which included the surface response technique. The mechanical properties of the specimens increased as the flow rate and layer thickness increased and the nozzle temperature decreased. The optimized printing parameters for the ranges used in this study include 110% flow rate, 140 μm layer thickness, and 240 °C nozzle temperature, which resulted in sintered parts with a tensile strength of 513 MPa and an elongation at break of about 60%.

## 1. Introduction

The fourth industrial revolution’s technical advancements (Industry 4.0) have resulted in substantial changes from the well-established Mass Production to the innovative Mass Customization. In this context, additive manufacturing (AM) or 3D-printing is a noteworthy production approach, as it enables Mass Customization’s major needs, namely high production rates and product customization [1]. AM is endowed with numerous advantages, i.e., freedom of design, waste minimization, fast prototyping and products customization, and includes numerous production processes and technologies depending on the type of printed material [2,3].

The possibility of tailoring product properties depending on consumer needs is of paramount importance in some industrial sectors such as the aerospace [4], biomedical, surgical and prosthetic industries [5,6,7]. All these fields extensively use metal components and even if several technologies are available for metal 3D-printing, i.e., selective laser melting (SLM), electron beam melting (EBM), direct metal laser sintering (DMLS), etc. [8], they are not competitive on the market due to the high investments required to purchase the printing apparatus. Due to the particular printing conditions, such as vacuum or inert atmosphere, and the high energy sources required, a metal 3D-printer can cost up to 1 M$ according to the data provided by Markforged [9], and its use is justified only in a limited number of fields where a reasonable return on investment is possible.

The use of polymer AM techniques in conjunction with well-established debinding and sintering (D&S) processes is a good solution to avoid these issues and give unique value to consumers in a timely manner. The green component printed with a polymeric binder highly filled with metal powders is subjected to the debinding/sintering process to remove the binder and achieve a dense metal object. In the aerospace, automotive, and medical device industries, these processes can be optimized and automated for high quality manufacturing [10]. In fact, in metal injection molding (MIM) technology, the debinding process is a critical step that affects the entire MIM process and, ultimately, the end product quality [11,12]. Polymer AM methods are much more cost-effective than metal ones requiring an investment cost between 15,000 and 100,000 $ according to the data reported by Formlabs [13]. Among them, fused deposition modeling (FDM) is the cheapest one [14] and this encouraged the investigation of the feasibility of the two-step process FDM printing/D&S cycle to produce dense metal parts [15,16].

Gonzalez-Gutierrez et al. [17] studied the feasibility of this two-step process for a 17-4 PH filled filament and the investigation of the resulting tensile properties disclosed an average Young’s modulus of 196 GPa, a tensile strength of 696 MPa and a strain at break of 4%. Kurose et al. [18] evaluated the influence of printing direction on the tensile properties of a 316L stainless steel proving that the flatwise printed samples were characterized by the highest tensile strength of 453 MPa, whereas the upright printed ones were characterized by the worst performance with a tensile strength of 100 MPa. Similar results were reported by Tosto et al. [19] who studied the tensile behavior of 316L and 17-4 PH stainless steel samples always printed in upright and flatwise directions. An increase of 23.2% in the yield strength, of 53.5% in the tensile strength and of 25.4% in the Young’s modulus were observed for 316L samples moving from the upright to the flatwise configuration, while increases of 7%, 1% and 5% were observed for the same parameters in the case of 17-4 PH.

Despite the positive results, several investigations have found that sintered parts made using metal FDM have inferior mechanical properties than those made with classic metal AM methods such as SLM/DMLS [20,21,22] and laser powder bed fusion (LPBF) [23]. Internal voids and polymeric binder residues acting as stress intensifiers are always blamed for poor mechanical performance, stressing the necessity to optimize process parameters for better mechanical results. The different processing processes involved (printing, debinding, and sintering) should be addressed in this optimization technique, with a focus on the final microstructure and mechanical properties. Godec et al. [24] recently demonstrated that the printing step is critical for achieving acceptable mechanical characteristics of metal specimens, and their work on 17-4 PH stainless steel highlighted the importance of the flow rate multiplier’s tensile properties. Thompson et al. [25] performed a detailed optimization of the debinding and sintering steps in order to maximize the mechanical properties of AISI 316L metal components, reaching a sintered component density of more than 95%. Singh et al. [26] optimized the entire production process for a Ti-6Al-4V, discovering the optimal operating parameters for both the printing and D&S processes, proving the critical role of the flow rate multiplier in the printing step and powder dimensions in the sintering process. The importance of material sensitivity to printing parameters, always for a Ti-6Al-4V, was also highlighted by Shaikh et al. [27] who estimated the process outcome in response to variable inputs and the relative sensitivity through a FEA-based thermomechanical process simulation, and in a further study developed a patient-specific maxillofacial implant prototype applying FDM and D&S to Ti-6Al-4V [28]. Previous research has highlighted the importance of systematically disclosing appropriate printing settings, a problem that has received limited attention in the literature, particularly for austenitic stainless steel AISI 316L.

The current study attempts to improve the mechanical properties of a sintered 316L stainless steel 3D-printed by FDM with a commercially available BASF Ultrafuse 316L filament. Currently, the knowledge about the impact of the chosen printing parameters on the mechanical behavior for this type of material is still limited by their recent introduction on the market. The current study aims to fill this gap improving the understanding using rationalized design of experiment (DoE) approach which can unveil all the effects. In a 2^3^ replicated screening design, the optimization process was based on the DoE technique, which took into account three main printing parameters: nozzle temperature, layer thickness, and flow rate. The FDM/D&S manufactured samples were tested in tension and bending, and the results were analyzed to determine the statistical significance of each printing parameter, and the best printing conditions were determined using response surface method. Furthermore, metal parts show anisotropic shrinkage following debinding and sintering (D&S), and manufacturing fully dense green parts could provide a prediction and mitigation of shrinkages in brown and sintered parts. For this reason, the present study also aims to unveil which printing parameters are mostly significant to obtain green parts characterized by the lowest presence of internal voids and therefore by lower shrinkage issues during the sintering phase. AISI 316L steel was selected as it represents a material of choice in biomedical devices due to the favorable combination of mechanical strength, cost effectiveness, ductility, and corrosion resistance [11].

## 2. Materials and Methods

### 2.1. Sample Production

The dense metal samples were produced using a combination of FDM, debinding, and sintering techniques. The green samples were 3D-printed using a desktop-size Zortrax M200 3D printer with a 0.4 mm bronze nozzle and BASF’s commercial metal-polymer filament Ultrafuse 316L. The filament has a nonslip surface and is filled with 316L metal particles, making it suitable for use in any Bowden or direct-drive extruder. To get the dense metal samples, BASF recommended outsourcing the green samples to a D&S service for postprocessing. For the Ultrafuse 316L, all printing settings were adjusted within the ranges indicated by BASF in the technical data sheet. To increase the print density of the green parts and sintered samples, three main printing parameters were identified: nozzle temperature, layer thickness, and flow rate. As a result, these are the printing parameters that were changed in the 2^3^ full factorial design to optimize the density and mechanical properties of green and sintered samples.

Multiple process variables are involved in FDM. These variables can be categorized into four groups, according to Agarwala et al. [29]: operation-specific variables, machine-specific variables, material-specific variables, and geometry-specific variables. To produce high-quality parts with good strength, concurrent optimization of these interconnected parameters is necessary [30].

Table 1 shows the printing settings that were kept constant throughout all printing setups, whereas Table 2 shows the low and high levels of the three parameters that were used in the full factorial design. The height of each layer printed on top of another is known as the layer height. Increasing the layer height will result in a poor surface finish and cause a staircase effect on curved surfaces, while decreasing the layer height will lengthen printing time as the component’s total number of layers grows [31]. In this study, the low level of layer height corresponds to the minimum reachable by the slicing software Z-Suite version 2.24.0 of Zortrax (Olsztyn, Poland), that is 90 μm, while the high level, equal to 140 μm, corresponds to the next value settable by the software, in the range suggested by the producer [32] and used in the previously published work [19].

Additionally, it is advisable to use an extrusion multiplier because a slight over-extrusion will fill in the spaces between the extruded lines and produce a green body with a high density [25], so the investigated values were 100% (default value) and 110%.

Finally, also the temperature was changed, setting it at 240 °C for the low level, in accordance with the findings of a previous study [19], and 250 °C for the high level, for enhancing the interlayer bonding and reducing the formation of voids investigated in a previous study [33].

The eight different printing configurations presented in Table 3 resulted from the combination of the three factors varying on two levels, each with three replicates of samples for a total of 24 samples for each test condition. All green specimens were oversized in accordance with the results of a prior study [19] due to the shrinkage experienced by samples as a result of the D&S process.

Table 4 shows the standard dimensions required by ASTM E8 for tensile testing and ASTM D790 for flexural testing, as well as the green parts dimensions.

### 2.2. Sample Characterization

**Figure 1:**
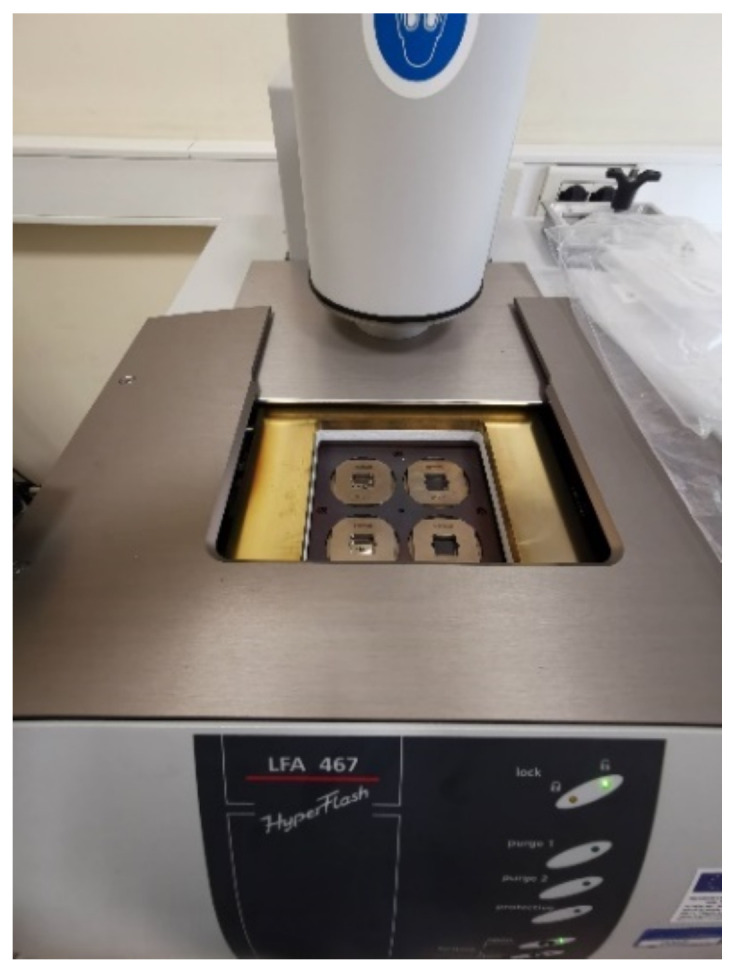
Thermal conductivity test setup on green parts.

Green samples were morphologically characterized to determine their density, porosity, and shrinkage as a result of the D&S process. Furthermore, the thermal conductivity of green samples was determined using ASTM E1461 tests on square samples with sides of 12.7 mm and a thickness of 2 mm. Equation (1) was used to calculate thermal conductivity:

(1) $$k=\alpha \cdot \rho \cdot C_{p},$$

where *α* is the thermal diffusivity (m^2^·s^−1^), *k* is the thermal conductivity (W·m^−1^·K^−1^), *ρ* is the density (kg·m^−3^) and *C_p_* is the specific heat capacity (J∙kg^−1^·K^−1^). The thermal diffusivity measurements were performed using a LFA 467 HT HyperFlash machine (NETZSCH-Gerätebau GmbH), and the material’s specific heat capacity (*C_p_*) was computed by differential scanning calorimetry (DSC), using a Mettler DSC1 Star System. The LFA Proteus analysis program processes the time and temperature data collected during the test in adiabatic conditions to determine the thermal diffusivity, and Equation (2) is used:

(2) $$\alpha =0.1388\frac{s^{2}}{t_{0.5}},$$

where *s* is the thickness of the sample and *t*_0.5_ is the time needed to increase the temperature by 50%. Once the values of *α*, *ρ* and *C_p_* have been calculated, the thermal conductivity values *k* are obtained by applying Equation (1). Before being tested, all of the samples were coated with graphite to eliminate light reflection errors, as the sample must not be translucent in the visible and near-IR wavelength ranges, and to maximize the samples’ absorption and emission capacity (Figure 1). Thermal conductivity experiments were conducted at temperatures ranging from room temperature to 90 °C, with measurements taking place in isothermal conditions at 30, 60, and 90 °C.

Sintered samples were subjected to a grain size analysis to evaluate the distribution of grain areas.

**Figure 2:**
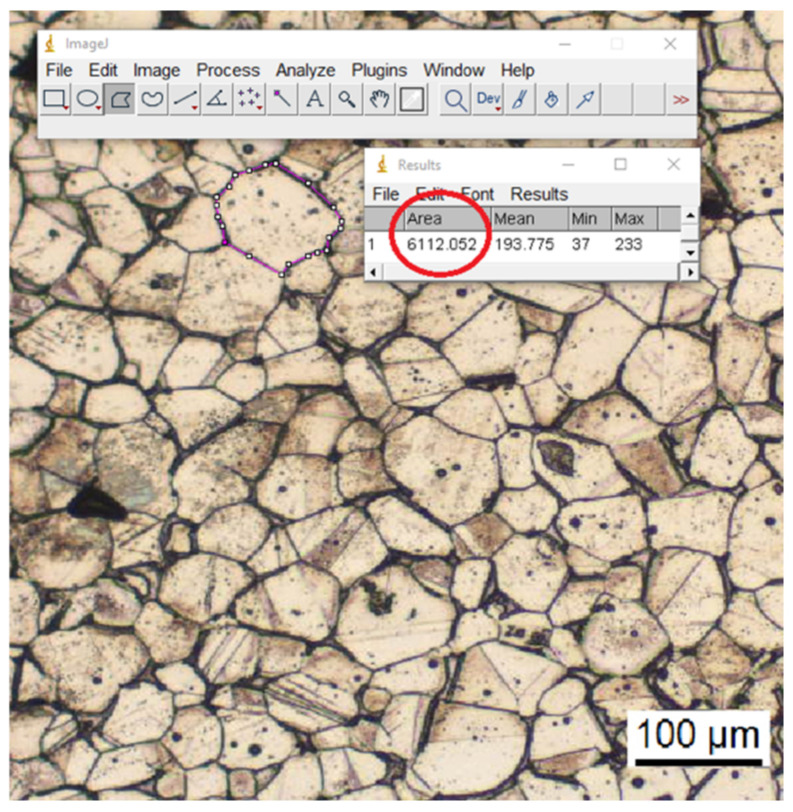
Procedure employed with the software Image J to evaluate grain areas.

After being lapped and polished, samples were exposed to electrochemical etching with oxalic acid diluted at 10% to highlight the grain boundaries. The treated samples were observed with an optical microscope (Leica DMI 5000, Leica Microsystems Srl, Buccinasco, Italy) and the resulting images were processed through the software Image J, version 1.52t (National Institutes of Health, Bethesda, MD, USA) in accordance with the procedure outlined in Figure 2 (grain area in the red circle).

Tensile and bending tests were also performed on dense metal samples. Tensile testing was performed in an Instron 5584 (Instron, Norwood, MA, USA) with a 100 kN load-cell in accordance with ASTM E8. The test parameters were as follows: 2 mm/min speed, 50 mm grip-to-grip separation, and accurate strain measurement using a high-resolution sensor arm extensometer with a gauge length of 30 mm. The specimen was also subjected to a 2 MPa preload. Flexural tests were performed using a Zwick/Roell Z010 (Zwick/Roell, Ulm, Germany) universal testing machine with a 10 kN load-cell in accordance with ASTM D790. The tests were carried out in a three-point bending configuration with a span length of 58 mm and a speed of 2 mm/min. A preload of 2 MPa was applied to the specimen in this case as well.

Finally, a surface fracture analysis of the tensile tested specimens was performed with a field-emission scanning electron microscope (FE-SEM) MIRA 3 by Tescan (Brno, Czech Republic) to disclose potential differences in densification, porosity and printed-structure among the various configurations.

### 2.3. Statistical Analysis

Statistical analysis included two different steps. At first, an inferential statistic was employed to determine whether the printing parameters selected as independent variables, i.e., nozzle temperature, layer thickness and flow rate, met the criteria for statistical significance proving to have an effect on the dependent variable, i.e., flexural and tensile modulus, yield and tensile strength. The F-test (ANOVA) was used to carry out the analysis and a *p*-value < 0.05 was adopted as a statistically significant limit meaning that a *p*-value lower than 0.05 confirms that the parameter under consideration had an actual effect on the dependent variable. This first part of the statistical analysis was performed through R-studio (Boston, MA, USA), an integrated development environment designed for the statistical computing programming language R, applying the linear model (lm) which also accounts for printing parameters interactions.

The second step included the implementation of the response surface methodology through a code specifically designed with the software Matlab^®^ version 2020a (The Math Works Inc., Natick, MA, USA). Considering that the system investigated in this study is overdetermined, the Least Squares Method was employed to determine the response surface. The rationale is to minimize the sum of the residuals *S* given in Equation (3):

(3) $$S=\sum_{i=1}^{N}e_{i}^{2},$$

where *e* is the residual given by the difference between the experimental values of the dependent variable obtained, *y_i_*, and the approximation function, *ŷ*(*x_i_*), which must be found:

(4) $$e_{i}^{\;}=y_{i}-\hat{y}\left(x_{i}\right).$$

The approximation function, i.e., the response surface, can be a linear model with or without interactions or a quadratic model. Based upon the results obtained in the first step of the statistical analysis, a linear model without interactions was selected in the present work as follows in Equation (5):

(5) $$Y\left(T,t,F\right)=a+bT+ct+dF,$$

where *Y* is the mechanical property under consideration, *T* the nozzle temperature, *t* the layer thickness and *F* the flow rate.

## 3. Results and Discussion

### 3.1. Green Parts Characterization

**Figure 3:**
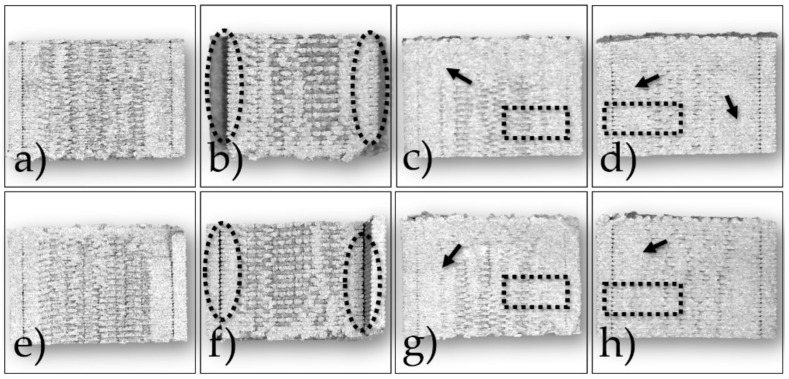
Optical images of green samples cross-sections printed according to the eight configurations in Table 3: (**a**) Nr. 5; (**b**) Nr. 4; (**c**) Nr. 1; (**d**) Nr. 2; (**e**) Nr. 7; (**f**) Nr. 8; (**g**) Nr. 6; (**h**) Nr. 3.

**Figure 4:**
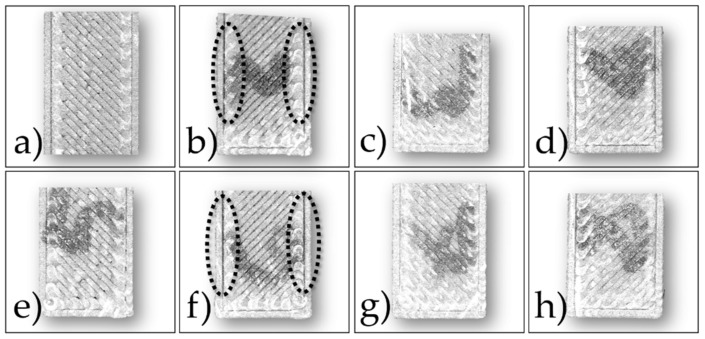
Optical images of green samples top surfaces printed according to the eight configurations in Table 3: (**a**) Nr. 5; (**b**) Nr. 4; (**c**) Nr. 1; (**d**) Nr. 2; (**e**) Nr. 7; (**f**) Nr. 8; (**g**) Nr. 6; (**h**) Nr. 3.

The green samples printed according to the configurations shown in Table 3 were analyzed at first by observing the cross-sections of the cryo-fractured samples and evaluating the presence of voids and incomplete bonding between the layers. In Figure 3 and Figure 4 are shown the cross-sections and the top surfaces of the eight configurations studied by optical microscopy, respectively.

In all cases, internal voids were detected across the sections, but some configurations show also delamination after fracturing. In particular, configurations 4 and 8 display a poor overlap between infill and outer walls (dashed ellipses in Figure 3b,f and Figure 4b,f). A similar issue can be observed for configurations 5 and 7 (Figure 3a,e and Figure 4a,e), even if it appears to be less evident. This lack of strong bonding is due to the low Flow rate, i.e., 100%, and to the absence, in the slicing software, of printing parameters such as overlap and connections between raster that reduce the internal voids. On the contrary, samples printed with the highest flow rate, i.e., 110%, show better-fused regions within the cross-sections (arrows in Figure 3c,d,g,h). However, some voids are still visible both in the internal and the outer areas (dashed rectangles in Figure 3c,d,g,h). According to these outcomes, the eight configurations can be grouped in two classes with similar internal morphologies depending on the flow rate used. Considering the class printed with the lowest flow rate, i.e., 100%, the two configurations printed with the highest layer thickness of 140 µm, i.e., 4 and 8, show samples with more defects with respect to those obtained with the lowest layer thickness of 90 µm, i.e., 5 and 7. Concerning the group with the highest flow rate of 110%, the differences among the internal sections of the four printing configurations are less significant.

### 3.2. Density and Shrinkage Evaluation of Sintered Parts

The results reported in the previous section are confirmed by the density and shrinkage evaluation of the sintered samples. Considering that the metal FDM sintered specimens are characterized by the presence of internal porosity, the bulk densities, *ρ_b_*, of the 24 bending samples were compared with the density of monolithic AISI 316 L (*ρ_AISI_* = 8000 kg/m^3^) to evaluate samples porosity, *p*, according to Equation (6):

(6) $$p=\left(1-\frac{\rho_{b}}{\rho_{AISI}}\right)\times 100.$$

The bending specimens were weighed in air and then again in water. The bulk density of the sintered parts, *ρ_b_*, was calculated according to Equation (7):

(7) $$\rho_{b}=\frac{w_{air}}{w_{air}-w_{liq}}\left(\rho_{L}-\rho_{0}\right)+\rho_{0},$$

where *ρ_b_* is the density of the sample, *w_air_* is the weight of the sample in air, *w_liq_* is the weight of the sample in water, *ρ_L_* is the density of water and *ρ*_0_ is the density of air. Specimens were weighed by using an analytic balance characterized by a resolution of 0.1 mg. The average densities and the average porosity of the sintered samples for each printing configuration are reported in Table 5.

Even in this case the configurations printed with the 100% flow rate, i.e., Nrs 4, 5, 7, 8, are characterized by the lowest density and the highest porosity whereas the 110% flow rate configurations display a porosity significantly lower than the 7.77% reported by Liu et al. [20] and the 5% reported by Thompson et al. [25] and by Quarto et al. [34]. These results are quite promising considering the relative density values reported for stainless steel 316L printed by binder jetting, i.e., 94% [35], 95–96% [36] and 97% [37], where powder bed packing fraction and homogeneity were deeply improved throughout the years by optimizing particles shape and distribution to achieve packing fraction higher than 70% [38].

Table 6 summarizes the average dimensions of the bending sintered samples measured by a caliper with an accuracy to the second decimal place. The actual shrinkages after D&S are also included in Table 6 next to each dimensional value. They were calculated according to Equation (8):

(8) $$Sh=1-\frac{L_{s}}{L_{g}},$$

where *Sh* is the shrinkage experienced by the specimen, *L_s_* and *L_g_* are the dimension of the sintered and green parts under consideration, respectively. All printing configurations display a shrinkage between 17% and 19% which is comparable with the 15–17% reported by Kurose et al. [15] and the 17% reported by Liu et al. [20] and is lower than the 20% reported by Thompson et al. [25]. These results are also comparable with those reported by Lecis et al. [39], i.e., 14.13–20.05%, who assessed the effect of different layer thickness and binder saturation values for a binder jetted 316L. The shrinkage observed does not differ much from the theoretical shrinkages expected in the slicing step at the time of choosing the oversizing factor (*OFS*), which can be calculated according to Equation (9):

(9) $$OFS=\frac{1}{1-Sh}=\frac{L_{g}}{L_{s}}.$$

Indeed, the *OFS* values of the present work were set equal to 20% on the xy plane and to 24% on the *z* axis, and they correspond to the expected theoretical shrinkage values of 17% on the xy plane and of 19% on the *z* axis.

### 3.3. Green Size Analysis of Sintered Parts

**Figure 5:**
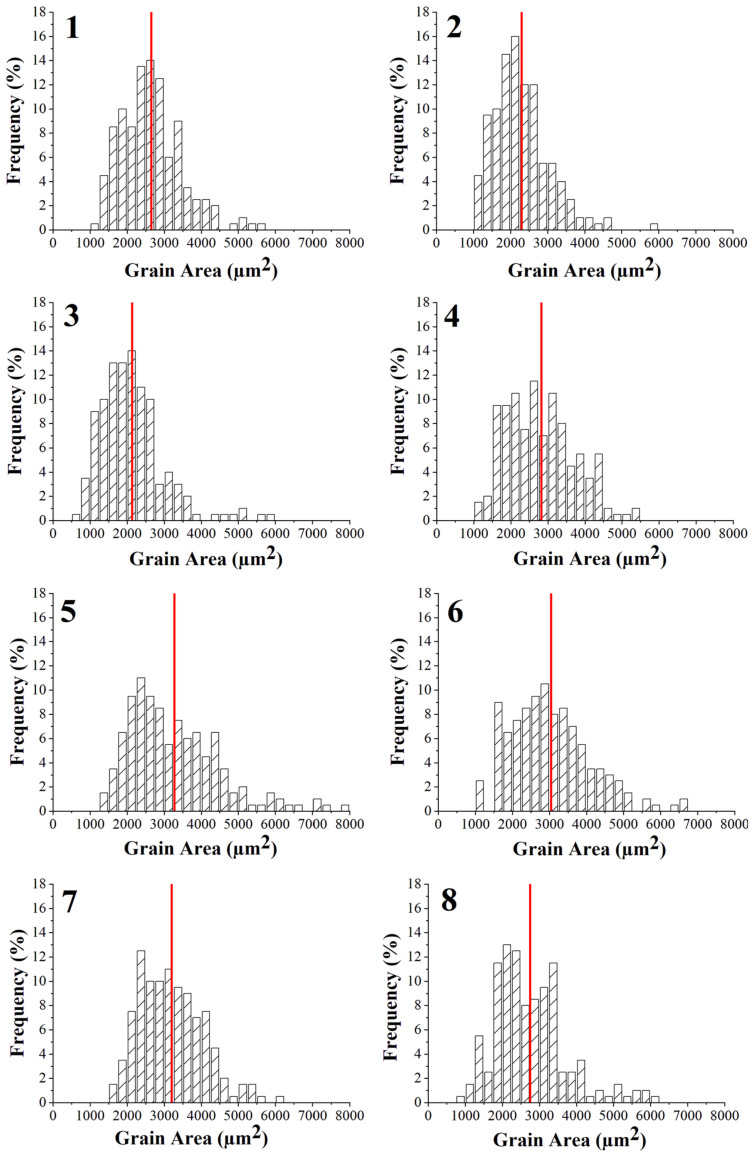
Grain area distributions of the eight printing configurations considered (the numbers at the top left of the graphs stand for the number of printing profiles investigated, as numbered in Table 3).

The grain size distributions of the eight printing configurations were evaluated and are reported in Figure 5, while Table 7 summarizes the average grain area of each group. All samples are characterized by equiaxed grains, as already acknowledged by Gong et al. [21] for the same technique, but also by Nastac et al. [37] for 316L produced by binder jetting. The equiaxed structure is the result of the low thermal gradients during the sintering process as also confirmed by Lee et al. [40] and by Gockel and Beuth [41] who were able to tailor the grain structure from a totally columnar to a totally equiaxed structure, by changing solidification rate and thermal gradient in an EBM process for an Inconel 718 alloy and of a Ti-6Al-4V, respectively. The specimens produced with the high-level flow rate, i.e., 110%, display a lower average grain area and a narrower distribution which tends to concentrate around the mean value. This can be ascribed to the higher amount of metal powder in the green sample, resulting from the higher flow rate, and therefore to the higher number of nucleation sites available. Another fundamental parameter is the layer thickness, in fact being equal the flow rate and the nozzle temperature, the specimens manufactured with the high-level thickness layer, i.e., 140 µm, are always characterized by an average grain area lower than the specimens produced with the 90 µm layer thickness, i.e., 2 < 1, 3 < 6, 4 < 5, 8 < 7. This can be ascribed to the fact that a lower layer thickness implies a higher number of discontinuities and therefore a higher surface area and a higher free energy of the system. To reduce system free energy, close the discontinuities and reach material densification, a major particle coalescence is required, thus determining an increase in the average grain area.

In particular, grain diameters range between 52 μm and 65 μm and these values agree with those reported by Nastac et al. [37] for 316L produced by binder jetting which ranged between 30 μm and 60 μm.

### 3.4. Mechanical Characterization and Statistical Analysis of Sintered Parts

**Figure 6:**
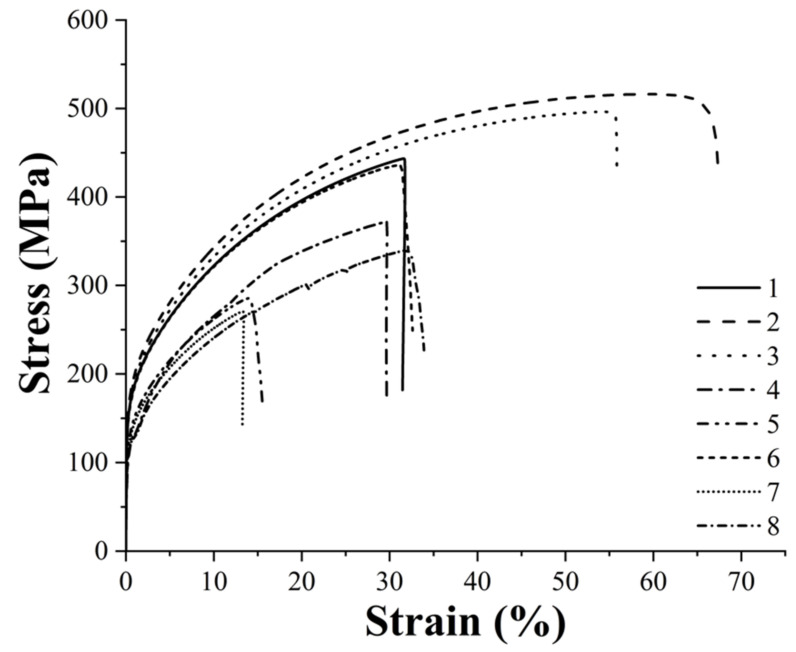
Typical tensile curves of the eight printing configurations under consideration (the numbers in the legend stand for the number of printing profiles investigated, as numbered in Table 3).

**Figure 7:**
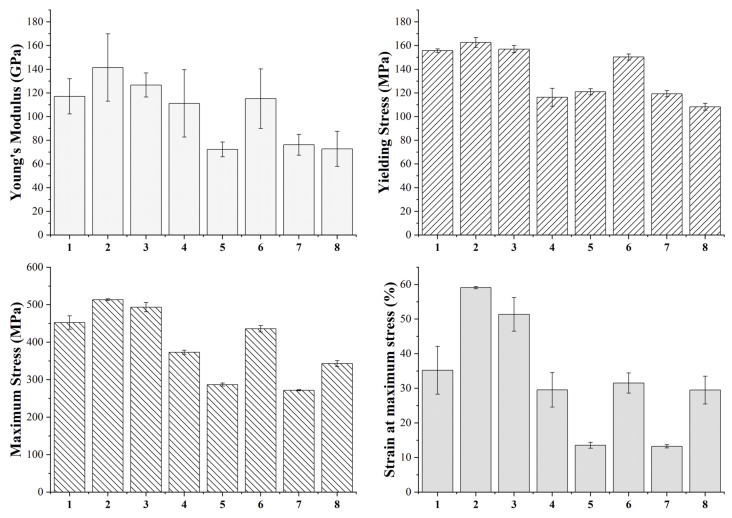
Tensile modulus, yield and tensile strength and strain at maximum strength of the eight printing configurations under study.

The specimens produced with the eight printing configurations reported in Table 3 were subjected to tensile and flexural tests to evaluate the effects of each printing parameter on the mechanical performance of the sintered samples. Figure 6 shows the typical tensile curves of each group and Figure 7 summarizes the tensile modulus, yield and tensile strength and strain at maximum strength values of each class. Moreover, the statistical significance of the different parameters and of their interaction is also reported in Table 8, Table 9, Table 10 and Table 11 for tensile modulus, yield and tensile strength and strain at maximum strength values, respectively.

**Figure 8:**
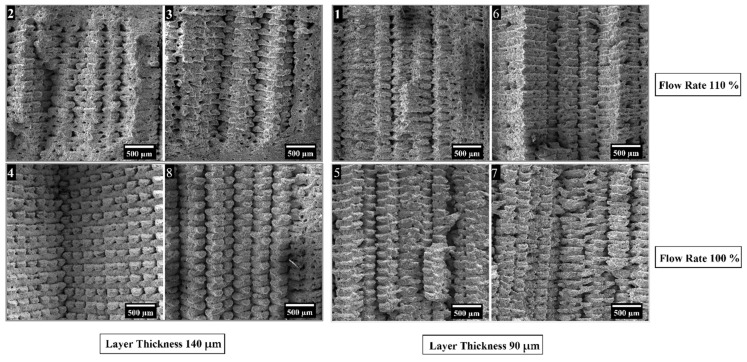
Surface fracture analysis of the eight printing configurations under study (numbers refer to the printing configurations as listed in Table 3).

The main parameter affecting the tensile properties of the printed specimens is the flow rate as also pointed out by the statistical analysis, which confirms the statistical significance of its effect for each property considered. In particular, all samples produced with the high-level flow rate of 110%, i.e., printing profiles nr. 1, 2, 3, 6, are characterized by a higher stiffness, a higher yield strength and a higher tensile strength. This must be ascribed to the higher density and the lower porosity of these sintered specimens, as confirmed by the data in Table 5, which ensure a higher microstructural homogeneity. The higher degree of densification of the 110% flow rate samples can be also observed from the FE-SEM micrographs of the fracture surface reported in Figure 8.

Concerning the other two printing parameters, the statistical significance of their effects is less pronounced with respect to flow rate, but they definitely play a significant role on the tensile strength of the material and some conclusions can be drawn. Focusing on the 110% flow rate specimens, it is possible to observe an increase in all tensile properties moving from the 90 µm layer thickness to the 140 µm one, in fact the printing profiles 2 and 3 display a better tensile response than 1 and 6. As a result of the lower number of printed layers, the 140 µm samples are characterized by a smaller number of discontinuities across the section of the specimen that must bear the tensile load as also confirmed by the micrographs in Figure 8. Similar results were already acknowledged for the tensile properties of FDM 3D printed polymers such as ABS (acrylonitrile butadiene styrene) [42], PLA [42] and graphene reinforced PLA [43]. Moreover, both the effects of flow rate and layer thickness observed in this work for the sintered parts are in perfect agreement with the results reported by Godec et al. [24], who showed an increase in the tensile properties of the green samples of a 17-4 PH stainless steel for increasing flow rate and layer thickness.

Considering the 110% flow rate samples and comparing those with the same layer thickness, it is possible to observe that even nozzle temperature has a non-negligible effect on the tensile properties. In particular, a decrease in the printing temperature leads to an increase in the tensile properties, in fact samples belonging to configuration 2 display higher tensile performance than those of configuration 3 as it happens also comparing configuration 1 with configuration 6. These results contrast with those reported by Godec et al. [24], who found out a decrease in the tensile properties for decreasing printing temperatures. This can be explained considering that Godec and co-authors worked on green parts whereas in this work sintered samples were considered. For green specimens, a decrease in temperature can increase air gaps and can affect negatively the bond between two subsequent layers, but when working with sintered samples the lower printing temperature can induce higher shear stress during the extrusion process, as also reported by Ahn et al. [44] for stainless steel powder injection molding, and these residual stresses can act as a driving force during the sintering process.

Moving to the 100% flow rate samples, the trend reported for the 110% samples is confirmed, as the highest tensile properties are achieved with configuration 4 which is the one characterized by the high-level thickness layer and by the low-level nozzle temperature. Configuration 8 ranks just after configuration 4 displaying a tensile modulus and a yield strength comparable with configurations 5 and 7, but a much higher tensile strength and ductility. Even in this case the higher layer thickness plays a major role endowing configurations 4 and 8 with a much higher tensile strength with respect to configurations 5 and 7.

A higher flow rate and layer thickness not only allow us to improve specimen density and to reduce discontinuities, but they also ensure a lower grain size fundamental to improve material mechanical properties according to Hall-Petch equation, as also confirmed by Feaugas and Haddou [45], who validated the feasibility of this relationship for AISI 316L and Nickel fcc polycrystalline metals. This explains why configurations 2 and 3, which are characterized by the lowest average grain size as shown in Section 3.3, display also the best tensile properties.

The results obtained are quite promising considering that the best tensile properties achieved, i.e., tensile strength of 513.3 MPa and maximum strain of 59.9%, are significantly higher than those reported by Gong et al. [21] and by Kurose et al. [18], display an improved ductility with respect to the results reported by Damon et al. [23] and are perfectly comparable with those reported by Sadaf et al. [46], as confirmed by the data summarized in Table 12. Moreover, these tensile properties compare favorably with those reported by Optimim [47] for their optimized Metal Injection Molded (MIM) 316L and with those always reported for MIM by Yoon et al. [48], by Zhang et al. [49] and by Afian Omar et al. [50], as shown in Table 12.

**Figure 9:**
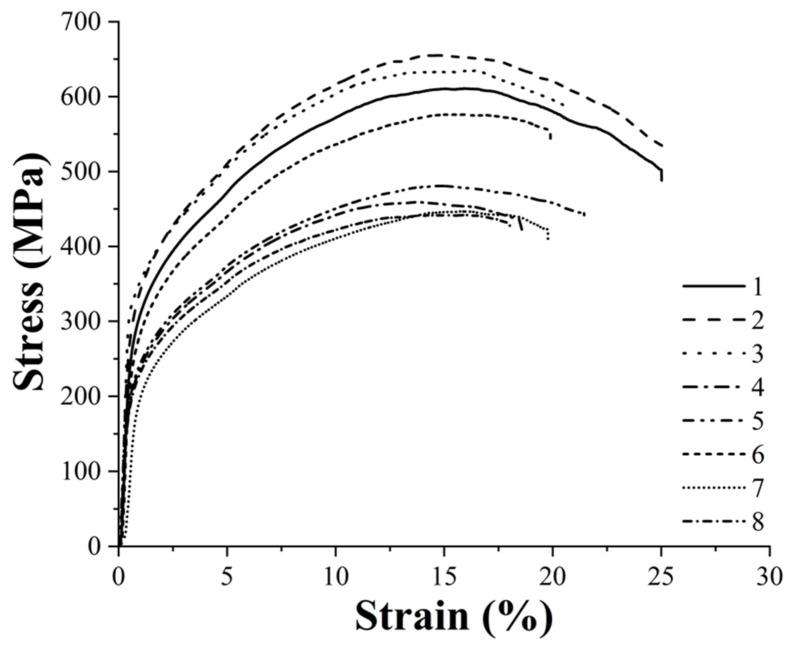
Typical bending curves of the eight printing configurations under consideration (the numbers in the legend stand for the number of printing profiles investigated, as numbered in Table 3).

**Figure 10:**
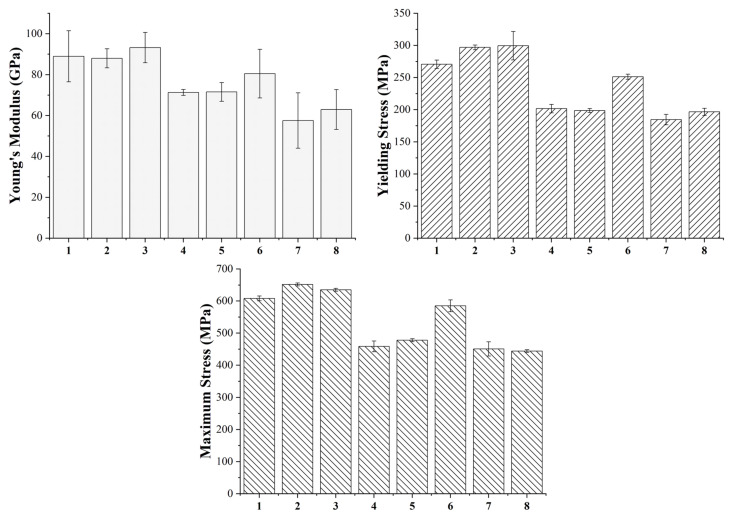
Flexural modulus, yield and flexural strength of the eight configurations under study.

The same analysis performed in tensile loading was also carried out in bending. Figure 9 shows the typical flexural curves of each group and Figure 10 summarizes the flexural modulus, yield and flexural strength values of each class. Even in this case, the statistical significance of the different parameters and of their interaction was evaluated and the results are reported in Table 13, Table 14 and Table 15 for the flexural modulus, yield and flexural strength values, respectively. As already acknowledged in tensile tests, flow rate is the most influential parameter in defining the bending properties of the samples, always exhibiting a strong statistical significance.

The four configurations with a 100% flow rate, i.e., 1, 2, 3 and 6, are characterized by the highest flexural stiffness and by the highest yield and flexural strength. Concerning the other two printing parameters, their significance is evident with regard to the strength values. In particular, the samples with a flow rate of 110% show the same trend as a function of layer thickness and nozzle temperature already acknowledged in tensile tests. Some variations with respect to the tensile behavior, however, can be noted for samples with 100% flow rate. In this case, layer thickness appears as a less influential parameter than nozzle temperature, in fact the samples printed at 240 °C, i.e., configurations 4 and 5, are characterized by a flexural strength and a flexural modulus that are higher than printing profiles 7 and 8. Therefore, also in this case, a decrease in printing temperature seems to play a favorable role in the mechanical properties.

**Figure 11:**
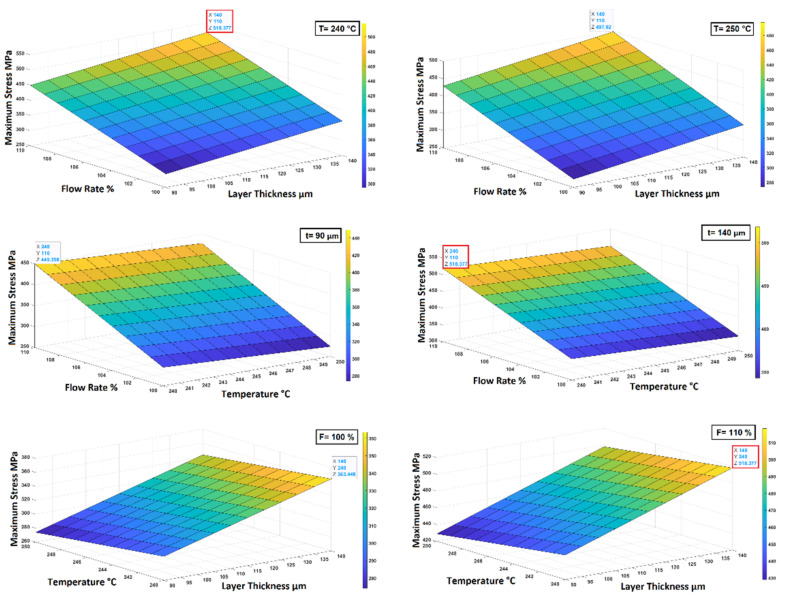
Surface response obtained for tensile strength.

Based upon all the results obtained and the analysis performed, the optimization process was concluded with the study and the evaluation of the surface response which allows us to correlate how the mechanical property under study varies as a function of the printing parameters. At first a quadratic approximation function was considered in the study to obtain the best possible fitting, but it was found out that the quadratic terms were null, so a linear approximation function was employed. Considering that the statistical significance of parameters interactions is negligible in most cases, a linear approximation function without interaction was considered. The surface response of each mechanical property was evaluated and was plotted by setting constant one of the parameters as shown in Figure 11 for the tensile strength.

Since the surface response tends to increase or decrease linearly with the printing parameters considered, the optimized configuration will be found in one of the extremes of the ranges considered. In particular, the best mechanical properties are achieved for a flow rate of 110%, a layer thickness of 140 µm and a nozzle temperature of 240 °C. These parameters coincide with the printing configuration 2 which is actually characterized by the highest tensile and bending properties, by the second highest density and the second lowest grain size area.

### 3.5. Thermal Conductivity Analysis of Green Parts

Configuration 2, the sintered component showing the best mechanical properties, was also selected to carry out the thermal conductivity tests on the green parts to evaluate if the related printing parameters are the best even from the green parts point of view. Moreover, flow rate proved to be the printing parameter that mainly influences sample density and mechanical properties, therefore printing configuration 4, which differs from printing configuration 2 only in the flow rate, was also selected to investigate directly the effect of the main printing parameter on thermal conductivity of green samples. The area of the analyzed samples was equal to 70%, the measurement was repeated three times, and the Cowen model was used for fitting the signal. The thermal conductivity values for the two configurations as a function of temperature are reported in Table 16.

It is possible to notice how the flow rate strongly influences the thermal conductivity of the green samples determining an increase higher than 40% moving from the low level (100%) of configuration 4 to the high level (110%) of configuration 2. This effect is certainly due to the greater amount of material added during printing, as well as to a better interlayer bonding between the slices, with a consequent reduction in the amount of air gaps and, therefore, an increase in the conductive properties.

## 4. Conclusions

The combination of FDM and the D&S process is a suitable cost-effective way to produce dense and customized metal parts. The present work aimed to optimize the printing parameters to improve the mechanical properties and the density of the sintered parts.

To reach this goal, three printing parameters were selected, i.e., nozzle temperature, layer thickness and flow rate, and a DoE analysis was performed through a 2^3^ full factorial design. The combination of the three factors varying on two levels, led to the eight different printing configurations. The specimens were tested in terms of tension and bending and the statistical significance of the effect of each printing parameter was assessed.

Flow rate proved to be the most critical value determining a significant increase in all mechanical properties and sample density and a decrease in the porosity and in the average grain size moving from a 100% to a 110%. Even layer thickness proved to be a significant parameter affecting material mechanical properties. In particular, an increase in layer thickness from 90 to 140 µm determined an increase in samples mechanical performance and a decrease in the average grain size being equal the flow rate and the nozzle temperature. Concerning nozzle temperature, a decrease in this parameter from 250 °C to 240 °C proved to be beneficial for the tensile and bending properties of the printed specimens.

The results obtained were supported by a surface response analysis which showed a linear increase or decrease of the mechanical properties under consideration for increasing printing parameters and enabled to select the printing configuration 2, i.e., flow rate 110%, layer thickness 140 µm and nozzle temperature 240 °C, as the optimum configuration. The latter is also characterized by the second highest density and the second lowest grain size area. Comparing the mechanical properties of the optimized samples with the results reported in the literature for this combined 3D-printing technique, an increase between 10–12% in the maximum tensile strength [18,21] and of 38–48% in the tensile strain at break [21,23] can be observed. Moreover, the tensile properties obtained are perfectly comparable with MIM ones [45,46,48] and the maximum tensile stress obtained is only 10% lower than that reported by Eshkabilov et al. [49], 15% lower than that reported by Brytan [52] and about 20% lower than that reported by Mertens et al. [53] for SLM printed samples. Lastly, it must be considered that the minimum tensile strength ensured by Atlas steel [54] and by United performance metals [55] for bulk AISI 316L is of 485 MPa that is 5.5% lower than that obtained in this work meaning that specimens obtained through this technique can become competitive on the market especially after the further optimization of the D&S process or the application of post-manufacturing treatments.

## Figures and Tables

**Table 1:** Common printing settings for the Ultrafuse 316L.

Printing Parameter	Unit	Value
Nozzle size	mm	0.4
Retraction distance	mm	1.5
Retraction speed	mm/s	45
Infill	%	100
Bed temperature	°C	90
Oversizing factor (xy)	%	20

**Table 2:** Printing settings varied in accordance with the experimental plan.

Factor	Symbol	Type	Unit	Low Level (−1)	High Level (+1)
Nozzle Temperature	A	Numerical	°C	240	250
Layer Thickness	B	Numerical	μm	90	140
Flow Rate	C	Numerical	%	100	110

**Table 3:** Printing profiles set according to the full factorial design.

Printing Profiles	Factors and Levels		
	Nozzle Temperature (°C)	Thickness (µm)	Flow Rate (%)
1	240	90	110
2	240	140	110
3	250	140	110
4	240	140	100
5	240	90	100
6	250	90	110
7	250	90	100
8	250	140	100

**Table 4:** Standard (ASTM E8 and D790) and green part dimensions for Ultrafuse 316L samples.

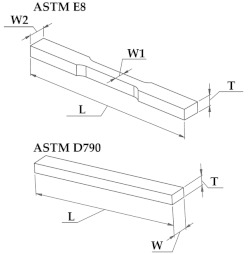	**Symbols**	**Standard Dimensions [mm]**	**Green Part Dimensions [mm]**
	**ASTM E8**		
	**L**	100	120
	**W1**	6	7.2
	**W2**	10	12
	**T**	6	7.44
	**ASTM D790**		
	**L**	80	96
	**W**	10	12
	**T**	5	6.20

**Table 5:** Average density and porosity of the bending samples.

Printing Profiles	Density (kg/m^3^)	Porosity (%)
1	7769.52 ± 16.73	2.88 ± 0.21
2	7752.69 ± 13.77	3.09 ± 0.17
3	7651.75 ± 22.26	4.35 ± 0.28
4	7013.55 ± 19.40	12.33 ± 0.24
5	7247.47 ± 18.10	9.40 ± 0.23
6	7447.90 ± 18.80	6.90 ± 0.23
7	6974.86 ± 9.18	12.81 ± 0.11
8	7166.93 ± 2.14	10.41 ± 0.03

**Table 6:** Dimensions and shrinkages of the sintered bending samples.

Printing Profiles	Length (mm)	Shrinkage	Width (mm)	Shrinkage	Thickness (mm)	Shrinkage
1	77.70 ± 0.1	19.06% ± 0.1	9.95 ± 0.01	17.08% ± 0.1	5.00 ± 0.01	19.35% ± 0.2
2	79.00 ± 0.1	17.71% ± 0.1	10.00 ± 0.01	16.67% ± 0.1	5.00 ± 0.01	19.35% ± 0.2
3	79.00 ± 0.1	17.71% ± 0.1	10.00 ± 0.01	16.67% ± 0.1	5.05 ± 0.01	18.55% ± 0.2
4	81.30 ± 0.1	15.31% ± 0.1	9.90 ± 0.01	17.50% ± 0.1	5.00 ± 0.01	19.35% ± 0.2
5	78.10 ± 0.1	18.65% ± 0.1	9.60 ± 0.01	20.00% ± 0.1	5.00 ± 0.01	19.35% ± 0.2
6	78.80 ± 0.1	17.92% ± 0.1	9.90 ± 0.01	17.50% ± 0.1	5.10 ± 0.01	17.74% ± 0.2
7	79.30 ± 0.1	17.40% ± 0.1	9.70 ± 0.01	19.17% ± 0.1	5.00 ± 0.01	19.35% ± 0.2
8	80.16 ± 0.1	16.50% ± 0.1	9.70 ± 0.01	19.17% ± 0.1	5.00 ± 0.01	19.35% ± 0.2

**Table 7:** Average grain area for the eight printing configurations (Red lines in Figure 5).

Printing Profiles	Average Grain Area (μm^2^)
1	2646.19
2	2298.25
3	2133.88
4	2820.65
5	3277.85
6	3041.90
7	3195.92
8	2749.26

**Table 8:** Statistical significance of the effects of the printing parameters and their interactions on samples tensile modulus.

	DF	Sum Sq	Mean Sq	F Value	Pr (>F)	
Temperature	1	984.3	984.3	1.7672	2.02 × 10^−1^	-
Thickness	1	1903.5	1903.5	3.4174	8.31 × 10^−2^	.
Flow	1	10,585.7	10,585.7	19.0046	4.87 × 10^−4^	***
Temperature:Thickness	1	1140.7	1140.7	2.0479	1.72 × 10^−1^	-
Temperature:Flow	1	117.6	117.6	0.2111	6.52 × 10^−1^	-
Thickness:Flow	1	0.1	0.1	0.0001	9.92 × 10^−1^	-
Temperature:Thickness:Flow	1	325.6	325.6	0.5846	4.56 × 10^−1^	-
Residuals	16	8912.1	557			

Signif. codes: [0–0.001] ‘***’ [0.05–0.1] ‘.’ [0.1–1] ‘-’.

**Table 9:** Statistical significance of the effects of the printing parameters and their interactions on samples tensile yield strength.

	DF	Sum Sq	Mean Sq	F Value	Pr (>F)	
Temperature	1	160.2	160.2	11.14	4.17 × 10^−3^	**
Thickness	1	1.5	1.5	0.1043	7.51 × 10^−1^	
Flow	1	9680.2	9680.2	673.503	1.67 × 10^−14^	***
Temperature:Thickness	1	16.7	16.7	1.1594	2.98 × 10^−1^	-
Temperature:Flow	1	0.7	0.7	0.0464	8.32 × 10^−1^	-
Thickness:Flow	1	322.7	322.7	22.4464	2.23 × 10^−4^	-
Temperature:Thickness:Flow	1	13.5	13.5	0.9391	3.47 × 10^−1^	-
Residuals	16	230	14.4			

Signif. codes: [0–0.001] ‘***’ [0.001–0.01] ‘**’ [0.1–1] ‘-’.

**Table 10:** Statistical significance of the effects of the printing parameters and their interactions on samples tensile strength.

	DF	Sum Sq	Mean Sq	F Value	Pr (>F)	
Temperature	1	2511	2511	30.3362	4.77 × 10^−5^	***
Thickness	1	28,581	28,581	345.3189	2.96 × 10^−12^	***
Flow	1	144,017	144,017	1740.015	<2.2 × 10^−16^	***
Temperature:Thickness	1	128	128	1.5462	0.23161	-
Temperature:Flow	1	28	28	0.3356	0.57044	-
Thickness:Flow	1	568	568	6.863	0.01858	-
Temperature:Thickness:Flow	1	48	48	0.5806	0.45717	*
Residuals	16	1324	83			

Signif. codes: [0–0.001] ‘***’ [0.01–0.05] ‘*’ [0.1–1] ‘-’.

**Table 11:** Statistical significance of the effects of the printing parameters and their interactions on samples tensile strain at maximum stress.

	DF	Sum Sq	Mean Sq	F Value	Pr (>F)	
Temperature	1	52.07	52.07	3.4147	8.32 × 10^−2^	.
Thickness	1	2164.67	2164.67	141.9647	2.28 × 10^−9^	***
Flow	1	3129.31	3129.31	205.2283	1.52 × 10^−10^	***
Temperature:Thickness	1	5.33	5.33	0.3495	5.63 × 10^−1^	-
Temperature:Flow	1	45.57	45.57	2.9884	1.03 × 10^−1^	-
Thickness:Flow	1	49.45	49.45	3.2431	9.06 × 10^−2^	-
Temperature:Thickness:Flow	1	6.77	6.77	0.4442	5.15 × 10^−1^	.
Residuals	16	243.97	15.25			

Signif. codes: [0–0.001] ‘***’ [0.05–0.1] ‘.’ [0.1–1] ‘-’.

**Table 12:** Comparison of the tensile properties achieved in the present work with those reported in previous works and other commercial production techniques.

Ref.	Production Technique	Tensile Strength (MPa)	Strain at Max Stress (%)
Present work	FDM + D&S	513.3	59.9
Gong et al. [21]	FDM + D&S	465	31
Kurose et al. [18]	FDM + D&S	453	-
Damon et al. [23]	FDM + D&S	500–520	32–37
Sadaf et al. [46]	FDM + D&S	521	-
Optimim [47]	MIM	517	50
Yoon et al. [48]	MIM	520	50–60
Zhang et al. [49]	MIM	460–560	29–58
Omar et al. [50]	MIM	510	-
Eshkabilov et al. [51]	SLM	574	49.6
Brytan et al. [52]	SLM	600	28
Mertens et al. [53]	SLM	659	16.6

**Table 13:** Statistical significance of the effects of the printing parameters and their interactions on samples flexural modulus.

	DF	Sum Sq	Mean Sq	F Value	Pr (>F)	
Temperature	1	244.04	244.04	2.8914	1.08 × 10^−1^	-
Thickness	1	106.64	106.64	1.2635	2.78 × 10^−1^	-
Flow	1	2862.57	2862.57	33.9167	2.59 × 10^−5^	***
Temperature:Thickness	1	140.6	140.6	1.6659	2.15× 10^−1^	-
Temperature:Flow	1	136.57	136.57	1.6181	2.22 × 10^−1^	-
Thickness:Flow	1	16.42	16.42	0.1945	6.65 × 10^−1^	-
Temperature:Thickness:Flow	1	24.22	24.22	0.287	6.00 × 10^−1^	-
Residuals	16	1350.4	84.4			

Signif. codes: [0–0.001] ‘***’ [0.1-1] ‘-’.

**Table 14:** Statistical significance of the effects of the printing parameters and their interactions on samples flexural yield strength.

	DF	Sum Sq	Mean Sq	F Value	Pr (>F)	
Temperature	1	477	477	5.3575	3.42 × 10^−2^	*
Thickness	1	3015	3015	33.861	2.61 × 10^−5^	***
Flow	1	42,588	42,588	478.2971	2.40 × 10^−13^	***
Temperature:Thickness	1	360	360	4.0473	6.14 × 10^−2^	.
Temperature:Flow	1	2	2	0.0229	8.82 × 10^−1^	-
Thickness:Flow	1	1335	1335	14.9934	1.35 × 10^−3^	**
Temperature:Thickness:Flow	1	63	63	0.7117	4.11 × 10^−1^	-
Residuals	16	1425	89			

Signif. codes: [0–0.001] ‘***’ [0.001–0.01] ‘**’ [0.01–0.05] ‘*’ [0.05–0.1] ‘.’ [0.1–1] ‘-’.

**Table 15:** Statistical significance of the effects of the printing parameters and their interactions on samples flexural strength.

	DF	Sum Sq	Mean Sq	F Value	Pr (>F)	
Temperature	1	2486	2486	15.976	1.04 × 10^−3^	**
Thickness	1	1726	1726	11.0911	4.24 × 10^−3^	**
Flow	1	157,976	157,976	1015.233	6.64 × 10^−16^	***
Temperature:Thickness	1	128	128	0.8195	3.79 × 10^−1^	-
Temperature:Flow	1	1	1	0.0075	9.32 × 10^−1^	-
Thickness:Flow	1	5398	5398	34.6915	2.28 × 10^−5^	***
Temperature:Thickness:Flow	1	17	17	0.1095	7.45 × 10^−1^	-
Residuals	16	2490	156			

Signif. codes: [0–0.001] ‘***’ [0.001–0.01] ‘**’ [0.1–1] ‘-’.

**Table 16:** Thermal conductivity values of the green parts for three values of observed temperatures.

	Thermal Conductivity [W·m^−1^·K^−1^]		
Printing Profiles	@ 30 °C	@ 60 °C	@ 90 °C
**2**	0.791 ± 0.001	0.807 ± 0.001	0.820 ± 0.001
**4**	0.562 ± 0.001	0.565 ± 0.001	0.568 ± 0.001
